# Genome Size Dynamics in Marine Ribbon Worms (Nemertea, Spiralia)

**DOI:** 10.3390/genes12091347

**Published:** 2021-08-28

**Authors:** Juraj Paule, Jörn von Döhren, Christina Sagorny, Maria A. Nilsson

**Affiliations:** 1 Department of Botany and Molecular Evolution, Senckenberg Research Institute and Natural History Museum Frankfurt, Senckenberganlage 25, D-60325 Frankfurt am Main, Germany; juraj.paule@senckenberg.de; 2 Institute of Evolutionary Biology and Ecology, University of Bonn, An der Immenburg 1, D-53121 Bonn, Germany; jdoehren@evolution.uni-bonn.de (J.v.D.); csagorny@evolution.uni-bonn.de (C.S.); 3 Senckenberg Biodiversity and Climate Research Centre, Senckenberganlage 25, D-60325 Frankfurt am Main, Germany; 4 LOEWE Centre for Translational Biodiversity Genomics (LOEWE-TBG), Senckenberganlage 25, D-60325 Frankfurt am Main, Germany

**Keywords:** C-value, genome size, flow cytometry, habitat, life history, ribbon worm, Nemertea, Lophotrochozoa, evolution, taxonomy

## Abstract

Nemertea is a phylum consisting of 1300 mostly marine species. Nemertea is distinguished by an eversible muscular proboscis, and most of the species are venomous. Genomic resources for this phylum are scarce despite their value in understanding biodiversity. Here, we present genome size estimates of Nemertea based on flow cytometry and their relationship to different morphological and developmental traits. Ancestral genome size estimations were done across the nemertean phylogeny. The results increase the available genome size estimates for Nemertea three-fold. Our analyses show that Nemertea has a narrow genome size range (0.43–3.89 pg) compared to other phyla in Lophotrochozoa. A relationship between genome size and evolutionary rate, developmental modes, and habitat was found. Trait analyses show that the highest evolutionary rate of genome size is found in upper intertidal, viviparous species with direct development. Despite previous findings, body size in nemerteans was not correlated with genome size. A relatively small genome (1.18 pg) is assumed for the most recent common ancestor of all extant nemerteans. The results provide an important basis for future studies in nemertean genomics, which will be instrumental to understanding the evolution of this enigmatic and often neglected phylum.

## 1. Introduction

Nemertea (ribbon worms) is a clade of worm-shaped animals comprising approximately 1300 species, with close phylogenetic affinities to Lophotrochozoa [1,2,3]. The species cover a large-size spectrum from several millimeters in interstitial species up to 30 meters in *Lineus longissimus* (Gunnerus, 1770) [4,5]. Most nemerteans are predators that hunt their prey with an eversible muscular proboscis that is housed in the rhynchocoel, which is a fluid-filled secondary body cavity situated dorsal to the intestine. The most species-rich lineage within Nemertea are Hoplonemertea that possess a proboscis that is armed with one large (Monostilifera) or several smaller (Polystilifera) calcareous stylets [6]. Another commonly encountered clade, Pilidiophora, is characterized by a unique, helmet-shaped larval type, the pilidium [7,8]. Within Pilidiophora, most species are known from the clade Heteronemertea [6]. Together, Hoplonemertea and Pilidiophora comprise a monophyletic lineage termed Neonemertea [9,10]. Palaeonemertea, as currently defined, represents a third clade comprising morphologically diverse species that is sister to Neonemertea [10,11,12].

Despite the relatively small number of species comprising Nemertea, there is considerable diversity with respect to ecology, reproductive biology, and development. The majority of nemertean species are benthic, with the most comprehensive distribution records being available for species occurring in shallow subtidal and intertidal to supralittoral, almost semi-terrestrial habitats [6]. Within Pilidiophora and Hoplonemertea, there are several freshwater species, whereas the few fully terrestrial species are restricted to the monostiliferan clade of Hoplonemertea [6]. Nemerteans are generally gonochoristic, but some hermaphroditic species exist, especially among the non-marine neonemerteans [13]. For sexual reproduction, the vast majority of nemertean species shed eggs into the surrounding sea water, which is followed by external fertilization. However, in several species, mucus spawning, internal fertilization, and viviparity have evolved [7,8,13,14]. The viviparous hoplonemertean species are assumed to show internal fertilization [13]. Very few species seem to have abolished sexual reproduction on a regular basis and largely reproduce by fissiparity, i.e., spontaneous fragmentation and subsequent complete regeneration of the fragments [15]. In most species, the fertilized eggs develop into free-swimming larvae that have to feed to proceed in their development [8]. The pilidium, the eponymous larval type of Pilidiophora, is such a pelagic, planktotrophic larval type [7,8,14]. In Hoplonemertea, the larval type has been termed decidula, whereas in palaeonemertean species, the larva has been termed planuliform larva [7,8,14]. In all major lineages, pelagic but non-feeding (lecithotrophic) larvae have evolved (reviewed in [14]). Furthermore, in Hoplonemertea and Pilidiophora, there are species in which no swimming larval stages are present, and development is confined to the clutch (intracapsular development) (reviewed in [7,8,14]). However, even within the clutch, the pilidiophoran species with intracapsular development have retained some characteristics of larval development also found in the free-swimming pilidium of their relatives [16]. Such intracapsular larvae of Pilidiophora have been termed Desor larvae and either feed on sibling eggs and embryos within the same clutch (ootrophic or adelphopagic, respectively), such as in *Lineus ruber* (Müller, 1774), or are truly non-feeding (i.e., lecithotrophic), such as in *Lineus viridis* (Müller, 1774) (reviewed in [7,8,14,16].

Variation in genome size is generated by three principal mechanisms: polyploidization, deletion or proliferation of DNA, and gain or loss of single chromosomes (i.e., aneuploidy). However, the universal controlling factors for the size of genomes across species are unknown, and different hypotheses concerning the mechanisms of genome size evolution have been postulated (e.g., [17]). Although several theories suggest neutral mechanisms such as population size influence (e.g., [18]) or mutational equilibrium due to the imbalance in indels (e.g., [19,20,21,22]), adaptive hypotheses of genome size evolution have also been considered [23,24]. Interestingly, altered genome sizes may have substantial consequences at a cellular, tissue, and organismal level and possibly influence metabolic and ecological features, which provides useful insights for the understanding of evolution and diversification (e.g., [20,25,26]).

The basic genomic characteristics of nemerteans, such as chromosome numbers, have been studied already since the end of the 19th century, but only a few data for a limited number of species have been accumulated since then (reviewed by [27]). In Heteronemertea, it was shown that there is considerable variation in chromosome numbers ranging from 2*n* = 4 to 2*n* = 56 showing signatures of polyploidy (e.g., *Lineus sanguineus* (Rathke, 1799)/*Lineus lacteus* (Rathke, 1843)) or aneu-/dysploidy (*L. ruber*) [27,28]. Moreover, certain variation in chromosome sizes was also demonstrated [27]. Therefore, it was surprising that the only study on genome size variation in Nemertea revealed relatively low variation (1C = 0.28–1.17 pg), even though species from all three major phylogenetic lineages were analyzed [29]. The highest genome size diversity within nemertean clades was documented for Heteronemertea covering four representatives of the polyphyletic genus *Micrura* Ehrenberg, 1828 [11,12,15]. Hoplonemertea was represented by early diverging *Nipponnemertes bimaculata* (Coe, 1901) and two species of *Paranemertes* Coe, 1901 in a more derived phylogenetic position [9]. The Palaeonemertea, which exhibits the most pronounced morphological diversity within Nemertea (e.g., [30] for spermatozoa; [30] for nephridia), was only represented by *Tubulanus polymorphus* Renier, 1804 from the Pacific coast of the USA [29]. Hence, the studied taxa covered only about 0.3% of the described nemertean diversity [1,3]. Moreover, the ecological and developmental diversity was not covered in detail, as all investigated species are found in mid-intertidal or subtidal habitats [29]. With two exceptions representing lecithotrophic larvae, *Micrura verrilli* Coe, 1901 and *T. polymorphus* [8,31], most of the other included species exhibit development with a feeding pilidium larva in Heteronemertea and a decidula larva in Monostilifera [8,32,33,34], although no information is published for *Paranemertes sanjuanensis* Stricker, 1982, *N. bimaculata,* and *Notospermus geniculatus* (Delle Chiaje, 1828) [29,35,36]. Nevertheless, a significant positive correlation was found between genome size and mid-range body length across the studied species [29], which is interesting, as long body size is typical in Nemertea (e.g., *L. longissimus*, see [4]).

By adding new data, covering morphologically divergent lineages, such as Cephalotrichidae as well as known ecological and developmental variation of the phylum [14,37,38,39,40,41,42,43,44], we aimed to (1) analyze the distribution and potential evolutionary consequences of genome size variation in the phylum Nemertea. More specifically, within a novel phylogenetic framework, we aimed to (2) test the correlation of body length and genome size as well as (3) relate genome size to habitat and several functional and developmental traits.

## 2. Materials and Methods

### 2.1. Nemertean Species Selection

Nemerteans were collected in the vicinity of Concarneau and Roscoff, France, during several field trips from 2018 to 2020. A list of studied samples, geographic origin, and collection history is provided in Supplementary Table S1. After collection, the animals were kept in sea water from the locality and stored at 12 °C. In total, the genome sizes of 18 nemertean species were estimated (Table 1). This included four representatives of Paleonemertea, five representatives of Hoplonemertea, and nine heteronemerteans. Due to the few distinguishing outer morphological characters, the mitochondrial COI (cytochrome C oxidase subunit I) gene was sequenced for all specimens, or conspecifics from the same locality, and compared to published sequences (see Section 2.3). Species identified by COI barcoding as well as species identified by morphological characters are listed in Supplementary Table S1, together with the GenBank reference to the corresponding COI barcode. In addition, six morphological, ecological, reproductive, and developmental traits for studied taxa were extracted from published literature or personal observations and are given in Supplementary Table S2.

### 2.2. Genome Size Estimation

Genome size (2C-value, [45]) was estimated by flow cytometry using the Partec CyFlow Space (Partec, Münster, Germany) equipped with a green solid-state laser. Sample preparation followed the two-step Otto protocol [46] with an internal standard *Glycine max* cv. Polanka (2C = 2.50 pg, [47]) or *Pisum sativum* L. cv. Ctirad (2C = 9.09 pg, [48]). Nemertean tissue was mixed with the leaf tissue of the internal reference standard and homogenized with a razor blade in a Petri dish containing 1 mL of ice-cold Otto I buffer (0.1 M citric acid, 0.5% Tween 20, [46]). The suspension was filtered through a 42 μm nylon mesh and incubated for approximately 15 min at room temperature. The staining solution consisted of 1 mL of Otto II buffer (0.4 M Na_2_HPO_4_·12 H_2_O), β-mercaptoethanol (final concentration of 2 μL/mL), intercalating fluorochrome propidium iodide (PI), and RNase IIA (both at final concentrations of 50 μg/mL). Fluorescence was induced by a 30 mW green solid-state laser (532 nm) and fluorescence intensities of 10,000–15,000 nuclei per measurement were recorded. Sample/standard ratios were calculated from the means of the sample and standard fluorescence histograms, and only histograms with coefficients of variation <5% for the G_0_/G_1_ sample peak were considered. Two to nine replicate measurements of each sample were carried out on different days in order to account for between-day variation caused by random instrument drift and/or non-identical sample preparation. The 2C values in picograms were also converted to 1C values in base pairs (1 pg = 978 Mb [49]). Additional genome size estimates for nemerteans were excerpted from two publications [29,50], data from other representatives of Lophotrochozoa were extracted from the Animal Genome Size Database and multiplied by 2 in order to get 2C values [51], which were used for further statistical analyses. The genome size of *Cerebratulus lacteus* (Leidy, 1851) (1C = 1.40 pg) was not included due to missing details of the estimation method [52].

### 2.3. DNA Isolation, PCR, Sequencing, and Phylogenetic Reconstruction

DNA was isolated from flash-frozen individuals using the Qiagen DNeasy Blood & Tissue Kit according to the manufacturer’s instructions. The mitochondrial COI gene was amplified with the primers LCO1490 and HCO2198 [53]. The resulting PCR products were sequenced from both strands on an ABI sequencer 3730 DNA analyzer (Applied Biosystems, Foster City, USA) by the laboratory center of the Senckenberg Biodiversity and Climate Research Centre Frankfurt (SBiK-F) or by LGC Genomics (Berlin, Germany) with the primers used for the PCR. COI sequences of additional species were downloaded from GenBank and aligned with MUSCLE [54] in AliView v1.26 [55] and inspected manually. IQ-TREE v1.6.12 with the implemented ModelFinder was used to build a maximum likelihood (ML) phylogeny [56,57,58] in order to identify the closest relative for each species and reconstruct a phylogenetic tree. Due to the short length of the partial COI gene and deep divergences of the included species, ML analyses were carried out for each of the three nemertean groups separately. The resulting trees were inspected and used to manually build a consensus phylogeny that conforms to previously published hypotheses based on larger datasets [10,15]. The topology was rooted at Palaeonemertea and consistent with previous phylogenies [10].

### 2.4. Data Analyses

Statistical analyses were performed in R v4.0.2 [59], and data were visualized as boxplots. The relationship between chromosome counts and 2C values was assessed by Pearson’s correlation coefficient. Comparative analyses were performed in R using phytools v0.6-60 [60] and OUwie v2.3 [61] using the consensus nemertean phylogenetic tree as mentioned above. The differences in genome size among different lineages or different ecological and developmental types were assessed by Kruskal–Wallis test as well as phylANOVA in phytools. To determine whether rates of genomic characters’ evolution differ among nemerteans from different habitats or possessing different functional/developmental traits, the fit of two Brownian motion (BM) and five Ornstein–Uhlenbeck (OU) models was compared using OUwie. Both BM and OU models estimate the rate of stochastic motion (σ^2^). The OU process allows the trait to fluctuate around an optimum value (θ) in parameter space with a strength of attraction (α) toward that optimum, while BM allows the trait to move equally to any parameter space. Models BM1 and BMS assign single and multiple rates (σ^2^) of random drift. OU1 and OUM model single and multiple optima (θ) for different clades with a single α and σ^2^. The remaining models assume either multiple σ^2^ (OUMV), multiple α (OUMA), or both (OUMVA) among clades. When fitting models using OUwie, the starting value θ_0_ was dropped from the model and assumed to be distributed according to the stationary distribution of the OU process (default setting). The performance of each model was assessed by (1) confirming that the eigenvalues of the Hessian matrix were positive [61] and (2) checking that the estimated optima (θ) of traits were not outside a plausible range. Only models passing these criteria were retained. The best-fitting model was selected using AIC weights based on the sample size-corrected Akaike information criterion (AICc) using the function aic.w in phytools.

Relationships between genome size and body length and width were evaluated by multiple phylogenetic generalized least-squares (PGLS) [62]. The PGLS analyses were carried out using the R package caper v1.0.1 [63] with the λ value estimated by maximum likelihood. The ancestral states of 2C-value were reconstructed using the maximum likelihood estimation (function fastAnc) and visualized on the phylogenetic tree using the function contMap in phytools.

## 3. Results

The new genome size estimates of 18 Nemertea species are listed along with the nine published values (Table 1) [29,50]. The new estimates show that the 2C values in Nemertea range between 0.43 pg in *Emplectonema gracile* (Johnston, 1837) (Hoplonemertea: Monostilifera) and 3.89 pg in *Lineus acutifrons* Southern, 1913 (Pilidiophora: Heteronemertea).

**Figure 1:**
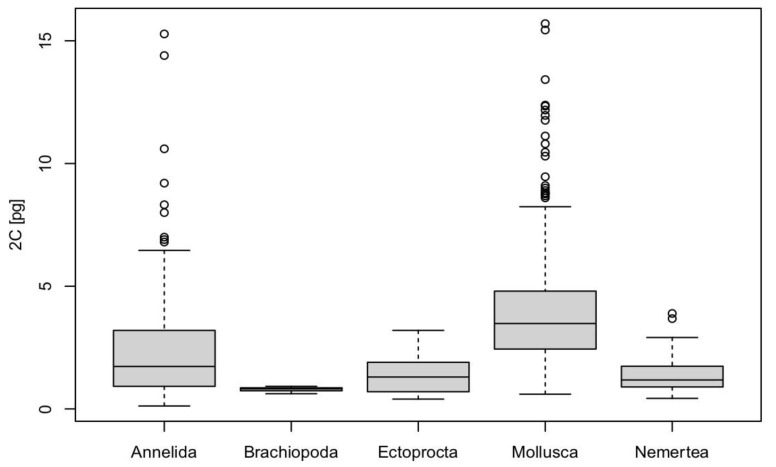
Boxplots of genome sizes across different lineages of Lophotrochozoa.

Compared to other representatives of Lophotrochozoa, Nemertea display a relatively narrow genome size range, about the same as Ectoprocta (Bryozoa), which range from 0.4 to 3.2 pg but are larger than in Brachiopoda (0.62 to 0.92 pg) (Figure 1). The 2C of the largest molluscan genome is 15.7 pg, whereas the 2C of the largest annelid genome is 15.28 pg.

**Figure 2:**
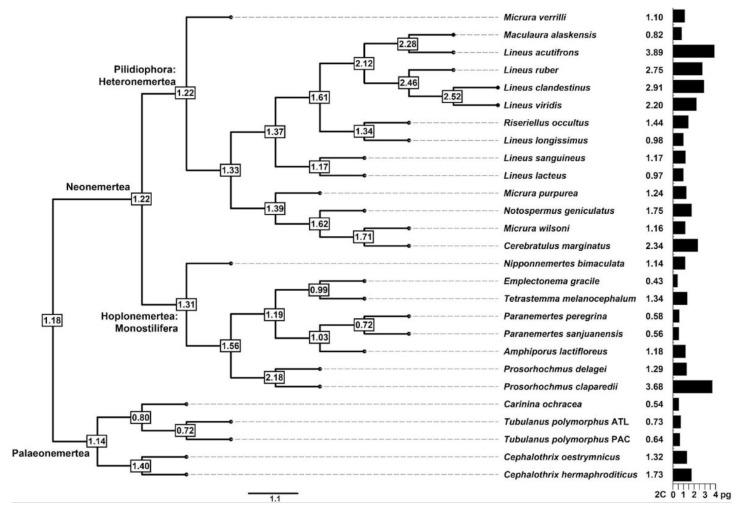
Phylogenetic tree of the studied samples and major nemertean lineages. Genome size (2C (pg)) is shown to the right of the tree as a black bar as well as in numerical form. Ancestral reconstruction of genome size for particular nodes is shown in white rectangles.

**Figure 3:**
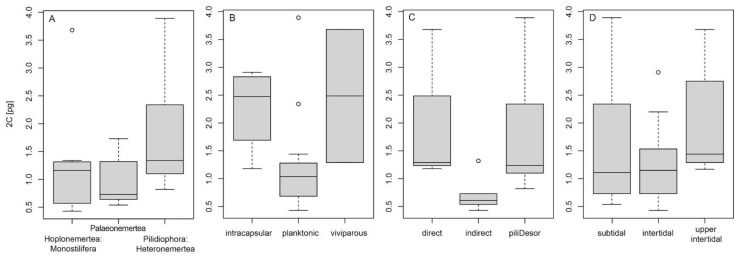
Boxplots of genome sizes (**A**): across studied lineages of Nemertea, (**B**): for different reproductive types (dev1), (**C**): for different developmental types (dev3) and (**D**): different habitats.

Within Nemertea, the largest genome size range was observed in Hoplonemertea, closely followed by Pilidiophora. Palaeonemertea exhibits the narrowest 2C interval ranging from 0.54 pg (*Carinina ochracea* Sundberg, Chernyshev, Kajihara, Kånneby, and Strand, 2009) to 1.73 pg (*Cephalothrix hermaphroditicus* (Gibson, Sanchez, and Mendez, 1990)) (Figure 2 and Figure 3). Nevertheless, as shown by a Kruskall–Wallis test as well as phylogenetic ANOVA, the differences observed among the nemertean lineages are not statistically significant (*p* = 0.156 and *p* = 0.813, respectively). Using fastANC, the ancestral 2C genome size for Nemertea was 1.18 pg (Figure 2).

When considering different ecological, reproductive, and developmental types (Supplementary Table S2), significant differences were revealed for the reproductive type (dev1) as well as the developmental type (dev3) using the Kruskall–Wallis test (*p* = 0.043 and *p* = 0.014, respectively) (Figure 3).

However, the significance of the differences was not confirmed by phylogenetic ANOVA. A weak negative correlation with chromosome number was revealed for the genome size (*r* = −0.462), although due to the very limited availability of chromosome counts for taxa studied here (*n* = 6, containing two outliers), the correlation was insignificant (*p* = 0.518). Regarding multiple PGLS regressions, none of the studied morphological characters (mean/max length and mean/max width) were significantly associated with genome size, even after removal of the outliers or log-transformation of the data.

When fitting multi-regime models of trait evolution, different models were suggested for different reproductive, developmental, and ecological traits (Table 2). For the reproductive type (dev1), an OUMV model was proposed as best fitting with higher genome size optima in viviparous and intracapsular developing species than taxa with planktonic larvae. The difference in the rate of stochastic motion was approximately by a factor of 10 among each of the three studied types with the intracapsular type bearing the lowest rate and the viviparous type bearing the highest rate. Concerning larval feeding mode (dev2), the OU1 model was revealed as best fitting, confirming a shared strength of attraction, rate of stochastic motion, as well as optimum genome size for both feeding types. The best fit of BMS for developmental type (dev3) suggested a circa 100 times higher rate of stochastic motion for genome-size evolution of direct and pilidium/Desor larval than for indirect development. Finally, the selection of the OUMV model for 2C evolution in different habitats revealed that the evolutionary rate (σ^2^) of genome size in subtidal and upper intertidal is 10 times higher than in the intertidal with different optima in all three habitats.

## 4. Discussion

### 4.1. Genome Size in Nemertea

So far, genome size estimates from the phylum Nemertea were known from only ten species and seven genera [29,50,52] out of the approximately 1300 extant species [1,3]. With our taxon sampling, we were able to increase the number of investigated nemertean species by 18. The coverage of the species-rich lineages Hoplonemertea and Pilidiophora is more than doubled, and members of the so far omitted cephalotrichid lineage of Palaeonemertea are included. Moreover, we were able to investigate the longest described nemertean species (*L. longissimus*) and species that are known to populate upper intertidal, almost semi-terrestrial habitats (*L. ruber* and *Prosorhochmus claparedii* Keferstein, 1862) [4,42,64]. Additionally, the diversity of developmental modes in the dataset is increased by including directly developing, such as *Amphiporus lactifloreus* (Johnston, 1828) [65] or viviparous, hermaphroditic species (*P. claparedii*), and species that have derived larval types (*L. ruber* and *L. viridis*) or are mainly reproducing asexually (*L. sanguineus*) [14,28,64].

### 4.2. Evolutionary Genome Size Dynamics

Due to the lack of a significant phylogenetic signal, our analyses revealed that the genome size is phylogeny-independent. Ancestral state reconstruction suggested multiple genome expansion and contraction events during the evolution of Nemertea. In Hoplonemertea: Monostilifera, one expansion (*P. claparedii*) and two contractions (independently in the species of *Paranemertes* and *E. gracile*) were observed. Given that the increase in genome size in closely related taxa usually indicates also an increment in the number of chromosomes (e.g., [66]), polyploidy in *P. claparedii* could be assumed, as the genome size is three times larger when compared to the closely related species *Prosorhochmus delagei* Oxner, 1907. In the species-rich Pilidiophora: Heteronemertea, there has been at least one (*Maculaura alaskensis* (Coe, 1901)) but maybe two additional genome contractions (independently in *L. longissimus* and *L. lacteus*) and three expansions (independently in *L. acutifrons*, *Cerebratulus marginatus* Renier, 1804, and the lineage of *Lineus clandestinus* Krämer, Schmidt, Podsiadlowski, Beckers, Horn, and von Döhren, 2016, *L. ruber*, and *L. viridis*). Interestingly, within our data, no signal for polyploidy among *L. sanguineus* and *L. lacteus* was revealed. Polyploidy in these two taxa could be assumed based on the previously published chromosome counts [27]. However, we could not properly assess the correlation of genome size and chromosome numbers in nemerteans due to the very limited availability of chromosome numbers for taxa studied here, and it is theoretically possible that even closely related taxa with different chromosome numbers bear similar genome sizes (e.g., [67]). In Palaeonemertea, two contraction events are suggested (independently in *C. ochracea* and the species of *Tubulanus* Renier, 1804), whereas one genome expansion seems to have occurred in *C. hermaphroditicus*. The possible reasons and drivers for the multiple independent changes in genome size of nemerteans are discussed below. However, it should be noted that there are limitations to ancestral character state reconstructions, as it can not reconstruct genome sizes that are larger or smaller than what is found in the extant species. 

### 4.3. Body Size and Genome Size

In several taxa, a significant positive correlation between genome size and body size was found (e.g., [68,69,70,71]). This was often explained by the generally positive correlation of genome size and cell size [72] and/or by the strength of the regulation of mitotic division (i.e., determinate growth) [69]. Using new as well as previously published data, no significant correlations of genome size and mean and maximum body length and body width were revealed in Nemertea. The longest described nemertean species, *L. longissimus* [4] for example, compares in genome size to significantly smaller species of the same clade, such as *M. alaskensis* [29,34]. On the other hand, smaller species such as *L. ruber* and *P. claparedii* have comparably large genomes, whereas the relatively large palaeonemertean species of the genus *Tubulanus* have very small genomes [29]. Moreover, the insignificant correlations were revealed also after removal of the body size outlier *L. longissimus* as well as after log-transformation of the data.

On the one hand, body size estimations in nemerteans are often difficult to assess, as these measures are mostly scored outside their natural habitat where they are rather fragile as well as due to their ability to extremely compress or stretch their bodies. Additionally, it has been demonstrated that species considerably grow even after they have reached sexual maturity; therefore, vastly disparate sizes in different adult specimens of the same species have been recorded e.g., in *Riseriellus occultus* Rogers, Junoy, Gibson, and Thorpe, 1993 [39]. Accordingly, the size ranges in literature are often rather broad (e.g., 14–80 mm in *L. ruber* [41,42]; 13–71 mm in *L. viridis* [42]). On the other hand, the relationships between genome size and body size, especially in large organisms, should be weaker, as distinct large body size might be more related to cell multiplication than to cell enlargement. Accordingly, we were not able to confirm the previously formulated indication of a positive genome and body size relationship [29]. Nevertheless, other morphological traits might be interesting to study in this context as e.g., genome size in crustaceans was shown to relate more to the size of propagules rather than to adult body size [73].

### 4.4. Life History and Ecological Traits and Genome Size

Our analyses showed that the evolution of genome size in Nemerteans is related to reproductive (dev1) and developmental type (dev3) as well as habitat, as differences in several trait evolutionary parameters were revealed.

Our data suggest that the mode of development in nemerteans influenced the evolutionary rate of genome size. Three different developmental modes were included in our dataset under developmental aspect (dev3), direct, indirect (planuliform/decidula larva), and pilidium/Desor-larva. Both direct development and development through pilidium/Desor larvae received about a 100 times higher rate of stochastic motion for genome size evolution than indirect development. Significant differences among developmental types were supported also by the Kruskall–Wallis test. Since indirect development represents the ancestral mode of development in Nemertea, while pilidium/Desor-larva and direct development are derived, the assumption of an elevated evolutionary rate in genome size evolution for the derived developmental modes is intuitive. Unexpectedly, no significant difference in genome size was detected for species with different larval feeding modes (dev2): feeding vs. non-feeding, i.e., lecithotrophic, the latter being the exclusive feeding mode of directly developing species. Thus, the allocation of additional maternal resources for the nourishment of the offspring in lecithotrophic development does not seem to be linked to genome size in the investigated species. The reasons for the significantly larger genomes of directly developing nemertean species (dev3: direct) remain puzzling. Comparative gene-expression studies might help to elucidate if larger genomes are caused by the addition of novel genes with specific functions in direct development or by accumulated non-coding genetic material. However, it has to be noted that all directly developing species also show intracapsular or viviparous development (dev1) and thus developmental variable dev3 might not be independent but just mirrors the effect of dev1 (see below: next paragraph).

An interesting pattern was exhibited for the relationship of developmental aspect (dev1: planktonic, intracapsular, viviparous) or habitat with genome size evolution. The differences between developmental types were significantly supported by the Kruskall–Wallis test. For both dev1 and habitat, three evolutionary optima were modeled, indicating the influence of different selective regimes on genome size to each of the three recorded parameter variants. For developmental aspect dev1, the highest evolutionary optimum was found in viviparous species, which was followed by species with intracapsular and planktonic reproduction. Likewise, the evolutionary optima for habitat revealed decreasing values from upper intertidal to subtidal. The most pronounced example in our dataset is *P. claparedii*, possessing the largest genome of the investigated hoplonemerteans occupying the most derived habitat (upper intertidal, almost semi-terrestrial). The intertidal heteronemertean species *L. ruber*, *L. viridis*, and *L. clandestinus* have considerably larger genomes than the majority of the closest relatives that dwell in lower intertidal or sublittoral habitats. These results can be straightforwardly interpreted in that the ancestral developmental mode (planktonic) and the ancestral habitat (subtidal) favor smaller genome size, whereas larger genome sizes are selected for in species that have derived developmental modes (intracapsular or viviparous) or live in abiotically more variable habitats (intertidal to upper intertidal).

However, the rates of genome size evolution in these two parameters seem somewhat counterintuitive, since they do not follow the same pattern as the respective evolutionary optima. For both parameters (dev1 and habitat), the most derived state (viviparous and upper intertidal, respectively) shows the highest values of stochastic motion, but in the allegedly ancestral states (planktonic and subtidal, respectively), the values of stochastic motion are not the lowest. Nemerteans with intracapsular reproduction have a 10 times lower rate than found in species with planktonic reproduction, and species found in upper intertidal and subtidal habitats have almost 10 times higher evolutionary rate compared to species living in intertidal habitats. Provided that these results are not statistical effects due to sample size but biological phenomena, a possible explanation could lie in effective population size. Due to the lower fecundity in species with intracapsular development or habitat limitations in the intertidal zone, the effective populations of the species could be smaller, which could result in lower genetic variation and, on a larger time-scale, in a lower rate of genome size evolution.

Genome size can change through the expansion of transposable elements (TE), which are genomic copies that are able to increase in numbers [74]. A direct correlation between genome size and the amount of TEs has been observed in some animal groups [75,76]. The mechanisms behind species-specific TE expansions are so far unknown. In some plant species (*Hordeum* L.), increased drought led to a stress-induced TE activity [77]. Whether the increased genome size optimum as well as higher evolutionary rate are mirroring the higher activity of TEs resulting from the colonization of less favorable semi-terrestrial habitat needs to be further investigated. Alternatively, an increase in genome size by means of polyploidy can be considered, implying the colonization of upper intertidal by polyploid lineages. Polyploidy has been repeatedly suggested for higher adaptive potential, which could be attributed to e.g., the increased genetic variability of polyploids, masking of mutations, gene redundancy, or heterosis (e.g., [78,79]). Based on our dataset, we cannot be fully conclusive about these results. However, we consider that this issue deserves further attention, especially due to the lack of terrestrial and symbiotic species covered in our data.

### 4.5. Nemertea and Genomic Biodiversity

The superphylum Lophotrochozoa includes six lineages: Annelida (17,733 species), Brachiopoda (392 species), Ectoprocta (=Bryozoa, 6,008 species), Mollusca (84,977 species), Nemertea (1358 species), and Phoronida (16 species) [1]. However, genome size estimates are known for about 500 lophotrochozoan species from five of these lineages, which is only about 0.45% of the known species [51]. The species-rich groups have the greatest genome size ranges, varying between 0.60 and 15.7 pg (2C) in Mollusca and 0.12 and 15.28 pg in Annelida. However, for both Mollusca and Annelida, there is currently a much higher number of genome size estimates available than in the other clades. Compared to other lineages in Lophotrochozoa, Nemertea displays a relatively narrow genome size range, about the same as Ectoprocta (0.2 pg to 1.6 pg), but larger than in Brachiopoda (0.31 to 0.46 pg).

Nemertea is one of the few invertebrate phyla with very limited published genomic data. So far, only one genome has been published [50]. Currently, large sequencing efforts are being directed to understand the genomic biodiversity with the aims of sequencing all life on earth [80]. Animal venoms are a potential source for drug development, medical research, and other applications [81]. As such, genome sequences from Nemertea coupled with proteomics and transcriptomics have the potential for identifying novel toxins and natural products [82,83]. Moreover, genome sequences will most probably be able to resolve controversies in nemertean phylogeny such as the Palaeonemertea monophyly, and on deeper timescales, the evolutionary radiation of Lophotrochozoa. Before starting a genome sequencing project, it is valuable to know the size of the genome or at least the range of possible sizes [84]. Hence, our genome size estimates provide valuable data for future genome sequencing projects of this neglected phylum. Due to the variation recovered in this study, future genome sequencing and comparative analyses should also focus on the role of repetitive regions or other phenomena in expanding or contracting genome size. For this, the identification of closely related species with strongly contrasting genome sizes such as *M. alaskensis*/*L. acutifrons* or *P. delagei*/*P. claparedii* are of key importance.

### 4.6. Remarks on Nemertean Taxonomy

In general, nemertean classification is somewhat chaotic with many genera being polyphyletic. This is especially true for Pilidiophora and likely due to the lack of distinguishing morphological characters [85]. The two genera *Lineus* Sowerby, 1806 and *Micrura*, for example, are non-monophyletic, as they are scattered throughout the heteronemertean phylogeny.

From the coast near Roscoff in France, two species of *Prosorhochmus* Keferstein, 1862 have originally been described: the fairly common species *P. claparedii* and the extraordinarily rare species *P. delagei* [64,86]. Based on morphological data, *P. delagei* was later synonymized with *P. claparedii* due to the absence of distinguishing diagnostic characters [87]. Since we found specimens that differ in both their genome size and the COI barcoding gene fragment, we conclude that besides the well-characterized *P. claparedii*, an additional *Prosorhochmus* species was found in Roscoff. Although only rarely found and never sampled for molecular markers before, we provisionally identify this second species as *P. delagei*.

The species *T. polymorphus* from the Pacific coast of North America is not identical with the species *T. polymorphus* collected at the Atlantic coast of Europe [88]. The locality of collection of the Atlantic species is closer to the type locality of *T. polymorphus*, and the Pacific species has originally been described as *Carinella rubra* Griffin, 1989. Therefore, the Pacific species should now be named *Tubulanus ruber* (Griffin, 1898). However, until a detailed revision of the species is published, we refer to either species as *T. polymorphus* Pacific (the species in [29]) and *T. polymorphus* Atlantic (the species dealt with herein).

## 5. Conclusions

The genome size estimates increase the available data for nemerteans three-fold and the phylum-wide genome size range increases from almost five-fold to over nine-fold. The results shed light on evolutionary and adaptive pressures in nemerteans. Trait analyses show that the highest evolutionary rate is found in upper intertidal, viviparous species with direct development, and the lowest evolutionary rate is found in intertidal species with intracapsular reproduction and indirect development with a planuliform or decidula larva potentially supporting the adaptive hypotheses of genome size evolution. Nemertean genome sequences will allow a deeper investigation of the role of TEs in genome size evolution. Future studies should extend the sampling with terrestrial and symbiotic nemerteans.

## Figures and Tables

**Table 1:** Genome sizes in Nemertea. Previously published 1C data by [29,50] were multiplied by two in order to get 2C. For the recalculation, the formula 1 pg = 978 Mbp by [49] was applied.

Species	Order:Class	Method	Standard	2C [pg]	±SD	1C [Mbp]	±SD	Source
*Amphiporus lactifloreus* (Johnston, 1828)	Hoplonemertea: Monostilifera	FCM	*G. max*	1.18	0.01	575.78	6.13	this study
*Carinina ochracea* [38]	Palaeonemertea: Tubulaniformes	FCM	*G. max*	0.54	0.00	264.88	1.03	this study
*Cephalothrix hermaphroditicus* Gibson, Sanchez, and Mendez, 1990	Palaeonemertea: Archinemertea	FCM	*G. max*	1.73	0.01	847.72	4.31	this study
*Cephalothrix oestrymnicus* Junoy and Gibson, 1991	Palaeonemertea: Archinemertea	FCM	*G. max*	1.32	0.03	646.34	12.24	this study
*Cerebratulus marginatus* Renier, 1804	Pilidiophora: Heteronemertea	FCM	*O. mykiss*	2.34	0.06	1144.26	29.34	[29]
*Emplectonema gracile* (Johnston, 1837)	Hoplonemertea: Monostilifera	FCM	*G. max*	0.43	0.01	209.75	5.42	this study
*Lineus acutifrons* Southern, 1913	Pilidiophora: Heteronemertea	FCM	*G. max*	3.89	0.13	1904.45	64.20	this study
*Lineus clandestinus* Krämer, Schmidt, Podsiadlowski, Beckers, Horn and von Döhren, 2016	Pilidiophora: Heteronemertea	FCM	*P. sativum*	2.91	0.10	1423.91	51.16	this study
*Lineus lacteus* (Rathke, 1843)	Pilidiophora: Heteronemertea	FCM	*G. max*	0.97	0.02	474.88	10.66	this study
*Lineus longissimus* (Gunnerus, 1770)	Pilidiophora: Heteronemertea	FCM	*G. max*	0.98	0.04	477.06	17.58	this study
*Lineus ruber* (Müller, 1774)	Pilidiophora: Heteronemertea	FCM	*P. sativum*	2.75	0.04	1342.41	19.59	this study
*Lineus sanguineus* (Rathke, 1799)	Pilidiophora: Heteronemertea	FCM	*G. max*	1.17	0.03	572.93	13.05	this study
*Lineus viridis* (Müller, 1774)	Pilidiophora: Heteronemertea	FCM	*P. sativum*	2.20	0.05	1074.02	25.35	this study
*Maculaura alaskensis* (Coe, 1901) (published as *Micura alaskensis*)	Pilidiophora: Heteronemertea	FCM	*O. mykiss*	0.82	NA	400.98	NA	[29]
*Micrura purpurea* (Dalyell, 1853)	Pilidiophora: Heteronemertea	FCM	*G. max*	1.24	0.02	606.21	9.81	this study
*Micrura verrilli* Coe, 1901	Pilidiophora: Heteronemertea	FCM	*O. mykiss*	1.10	NA	537.90	NA	[29]
*Micrura wilsoni* (Coe, 1904)	Pilidiophora: Heteronemertea	FCM	*O. mykiss*	1.16	NA	567.24	NA	[29]
*Nipponnemertes bimaculata* (Coe, 1901) (published as *N. bimaculatus*)	Hoplonemertea: Monostilifera	FCM	*O. mykiss*	1.14	0.02	557.46	9.78	[29]
*Notospermus geniculatus* (Delle Chiaje, 1828)	Pilidiophora: Heteronemertea	k-mer	NA	1.75	NA	859.00	NA	[50]
*Paranemertes peregrina* Coe, 1901	Hoplonemertea: Monostilifera	FCM	*O. mykiss*	0.58	0.00	283.62	0.00	[29]
*Paranemertes sanjuanensis* Stricker, 1982	Hoplonemertea: Monostilifera	FCM	*O. mykiss*	0.56	0.16	273.84	78.24	[29]
*Prosorhochmus claparedii* Keferstein, 1862	Hoplonemertea: Monostilifera	FCM	*G. max*	3.68	0.10	1801.70	50.97	this study
*Prosorhochmus delagei* Oxner, 1907	Hoplonemertea: Monostilifera	FCM	*P. sativum*	1.29	0.02	631.60	11.34	this study
*Riseriellus occultus* Rogers, Junoy, Gibson, and Thorpe, 1993	Pilidiophora: Heteronemertea	FCM	*G. max*	1.44	0.02	706.15	10.34	this study
*Tetrastemma melanocephalum* (Johnston, 1837)	Hoplonemertea: Monostilifera	FCM	*G. max*	1.34	0.04	657.40	18.19	this study
*Tubulanus polymorphus* Renier, 1804 (Atlantic)	Palaeonemertea: Tubulaniformes	FCM	*G. max*	0.73	0.02	358.47	11.80	this study
*Tubulanus polymorphus* Renier, 1804 (Pacific)	Palaeonemertea: Tubulaniformes	FCM	*O. mykiss*	0.64	0.08	312.96	39.12	[29]

**Table 2:** Parameters estimated using best-fitted models of trait evolution for reproductive type (dev1), feeding mode (dev2), developmental type (dev3), and habitat. α—strength of attraction towards optimum, σ^2^—rate of stochastic motion, θ—optimum value, S.E.—standard error.

Genomic Character/Model	Groups	α	σ^2^	θ (S.E.)
dev1/OUMV	intracapsular	0.349	0.004	2.973 (1.150)
	planktonic	0.349	0.039	0.975 (0.118)
	viviparous	0.349	0.513	5.609 (2.978)
dev2/OU1	non-feeding	0.219	0.165	1.106 (0.267)
	feeding	0.219	0.165	1.106 (0.267)
dev3/BMS	direct	N/A	0.631	1.314 (0.418)
	indirect	N/A	0.006	1.314 (0.418)
	pilidium/Desor larva	N/A	0.539	1.314 (0.418)
habitat/OUMV	subtidal	0.305	0.191	0.437 (1.394)
	intertidal	0.305	0.025	1.096 (0.097)
	upper intertidal	0.305	0.207	3.615 (1.093)

## Data Availability

Sequence data are deposited under accession numbers MZ558339-MZ558355 at NCBI GenBank (https://www.ncbi.nlm.nih.gov/genbank/ accessed 25 August 2021).

## Supplementary Materials

The following are available online at https://www.mdpi.com/article/10.3390/genes12091347/s1, Table S1: Collection history of studied accessions, Table S2: Morphological, ecological, reproductive, and developmental traits of studied nemertean taxa.
