# Supplementary material for: Genome Size Dynamics in Marine Ribbon Worms (Nemertea, Spiralia)
Source: Genes (Basel). 2021 Aug 28;12(9):1347. doi: 10.3390/genes12091347 (PMC8468679; doi:10.3390/genes12091347)
Supplement: Supplementary file 1 [file genes-12-01347-s001.zip › Paule_SupTable1_V1.pdf]

Supplementary Table S1: Collection history of studied accessions. Lat – latitutde, Long – longitude, Coll – collected by, COI – GenBank accession number of COI sequence used for barcoding/phylogenetic reconstruction, DetB – species identified primarily by COI barcoding, DetM - species identified primarily by morphological characters, SB – Station Biologique de Roscoff. Country codes follow ISO 3166-1 Alpha-3.

Species	Locality	Habitat	Lat	Long	Date	Time	COI	DetB	DetM
Amphiporus lactifloreus	FRA, Roscoff, beach in front of SB	mid-intertidal	48.727	-3.990	20.03.19	11.30-12.30	MZ558351		X
Carinina ochracea	FRA, Trégunc, Anse de Pouldohan	lower intertidal	47.849	-3.894	27.10.18	11.30-12.00	MZ558340	X	
Cephalothrix hermaphroditicus	FRA, Roscoff, beach in front of SB	mid-intertidal	48.728	-3.988	27.10.18	14.00-15.00	MZ558354	X	
Cephalothrix oestrymnicus	FRA, Trégunc, Pointe de la Jument	mid-intertidal	47.835	-3.902	25.03.19	13.45-14.00	MH681899		X
Emplectonema gracile	FRA, Le Cabellou, Plage du Large	mid-intertidal	47.854	-3.915	23.03.19	12.00-13.00	MZ558350		X
Lineus acutifrons	FRA, Trégunc, Anse de Pouldohan	lower intertidal	47.835	-3.902	10.01.20	10.30-11.30	MZ558339	X	
Lineus clandestinus	FRA, Roscoff, beach in front of SB	mid-intertidal	48.728	-3.988	09.01.20	11.00-12.00	MZ558346	X	
Lineus lacteus	FRA, Le Cabellou, Plage du Large	mid-intertidal	47.854	-3.915	27.10.18	10.30-11.15	MZ558347	X	
Lineus longissimus	FRA, Le Cabellou, Plage du Large	lower intertidal	47.854	-3.915	23.03.19	12.00-13.00	MZ558343	X	
Lineus ruber	FRA, Roscoff, beach in front of SB	upper intertidal	48.727	-3.990	27.10.18	14.00-15.00	MZ558344	X	
Lineus sanguineus	FRA, Le Cabellou, Plage du Large	upper intertidal	47.854	-3.915	27.10.18	10.30-11.15	MZ558348	X	
Lineus viridis	FRA, Roscoff, beach in front of SB	mid-intertidal	48.727	-3.990	27.10.18	14.00-15.00	MZ558345	X	
Micrura purpurea	FRA, Le Cabellou, Plage du Large	mid-intertidal	47.854	-3.915	10.01.20	09.00-10.00	MZ558341	X	
Prosorhochmus claparedii	FRA, Roscoff, beach in front of SB	upper intertidal	48.727	-3.990	27.10.18	14.00-15.00	MZ558355	X	
Prosorhochmus delagei	FRA, Roscoff, beach in front of SB	upper intertidal	48.728	-3.988	09.01.20	12.00-13.00	MZ558353	X	
Riseriellus occultus	FRA, Le Cabellou, Plage du Large	upper intertidal	47.854	-3.915	27.10.18	10.30-11.15	MZ558342	X	
Tetrastemma melanocephalum	FRA, Le Cabellou, Plage du Large	mid-intertidal	47.854	-3.915	21.02.19	14.00-15.00	MZ558352		X
Tubulanus polymorphus (Atlantic)	FRA, Roscoff, beach in front of SB	lower intertidal	48.728	-3.988	14.07.19	10:00-12.00	MZ558349	X	
