# Supplementary material for: Genome Size Dynamics in Marine Ribbon Worms (Nemertea, Spiralia)
Source: Genes (Basel). 2021 Aug 28;12(9):1347. doi: 10.3390/genes12091347 (PMC8468679; doi:10.3390/genes12091347)
Supplement: Supplementary file 1 [file genes-12-01347-s001.zip › Paule_SupTable2.pdf]

Supplementary Table S2: Morphological, ecological, reproductive and developmental traits of studied nemertean taxa. dev1-dev3 represent different coded reproductive/developmental features. Planktonic/intracapsular – development with/without free-swimming stage, viviparous – development within the mother; feeding/non-feeding – development with/without external food supply; indirect/direct – development with/without larva (not of the pilidium or Desor-larva type), piliDesor – with pilidium (planktonic) or Desor-larva (intracapsular). Further explanation of terms, see main text.

**Species**	**length [mm]**	**length mean [mm]**	**length max [mm]**	**width [mm]**	**width mean [mm]**	**width max [mm]**	**dev1**	**dev2**	**dev3**	**habitat**	**Reference**
Amphiporus lactifloreus	25-35-100	53.34	100	10-15-20-30	1.875	3	intracapsular	non-feeding	direct	intertidal	[41,65]
Carinina ochracea	25-45	47.5	45	1-1.5	1.25	1.5	planktonic	feeding	indirect	subtidal	[38,40]
Cephalothrix hermaphroditicus	150-200	175	200	1.5-2	1.75	2	NA	NA	NA	intertidal	[44]
Cephalothrix oestrymnicus	70	70	70	0.5	0.5	0.5	planktonic	feeding	indirect	intertidal	[37,43]
Cerebratulus marginatus	200-300 -1000	500	1000	25	25	25	planktonic	feeding	piliDesor	subtidal	[29,41]
Emplectonema gracile	500	500	500	3-4	3.5	4	planktonic	feeding	indirect	intertidal	[41]
Lineus acutifrons	150-170	160	170	5-7	6	7	planktonic	feeding	piliDesor	subtidal	[41]
Lineus clandestinus	11-48	42	71	1	1.5	2	intracapsular	non-feeding	piliDesor	intertidal	[42]
Lineus lacteus	600	600	600	1-2	1.5	2	planktonic	feeding	piliDesor	intertidal	[41]
Lineus longissimus	5000-15000 -30000	16666.7	30000	5	5	5	planktonic	feeding	piliDesor	subtidal	[41]
Lineus ruber	14-43-80	29.5	48	1-2-3	1	1	intracapsular	feeding	piliDesor	upper intertidal	[41,42]
Lineus sanguineus	100-200	150	200	2-3	2.5	3	planktonic	feeding	piliDesor	upper intertidal	[41]

Lineus viridis	13-71	45.67	80	1-2	2	3	intracapsular	non-feeding	piliDesor	intertidal	[42]
Maculaura alaskensis	20-50	35	50	NA	NA	NA	planktonic	feeding	piliDesor	intertidal	[29,34]
Micrura purpurea	200	200	200	2-3	2.5	3	planktonic	feeding	piliDesor	subtidal	[41]
Micrura verrilli	100-150	125	150	NA	NA	NA	planktonic	non-feeding	piliDesor	intertidal	[29]
Micrura wilsoni	150-200	175	200	2-3	2.5	3	planktonic	feeding	piliDesor	intertidal	[29,33]
Nipponnemertes bimaculata	40-60	50	60	NA	NA	NA	NA	NA	NA	intertidal	[29]
Notospermus geniculatus	60	50	60	2	2	2	NA	NA	NA	intertidal	[36]
Paranemertes peregrina	80-110	95	110	1-2	1.5	2	planktonic	non-feeding	indirect	intertidal	[29,32]
Paranemertes sanjuanensis	100-114-120	111.3	120	0.2	0.2	0.2	NA	NA	NA	intertidal	[29,35]
Prosorhochmus claparedii	35-40	37.5	40	1-2	1.5	2	viviparous	non-feeding	direct	upper intertidal	[41]
Prosorhochmus delagei	20-25	22.5	25	0.75-1	0.875	1	viviparous	non-feeding	direct	upper intertidal	[86,87]
Riseriellus occultus	700	700	700	1.0-1.3	1.15	1.3	planktonic	feeding	piliDesor	upper intertidal	[39,41]
Tetrastemma melanocephalum	30-60	45	60	2.0-2.5	2.25	2.5	NA	NA	NA	intertidal	[41]
Tubulanus polymorphus (Atlantic)	500	500	500	5	5	5	planktonic	feeding	indirect	subtidal	[41]
Tubulanus polymorphus (Pacific)	100-200	150	200	NA	NA	NA	planktonic	non-feeding	indirect	intertidal	[29]
